# Racial differences in cigarette brand recognition and impact on youth smoking

**DOI:** 10.1186/1471-2458-13-170

**Published:** 2013-02-25

**Authors:** Amanda L Dauphinee, Juliana R Doxey, Nina C Schleicher, Stephen P Fortmann, Lisa Henriksen

**Affiliations:** 1 Stanford Prevention Research Center, Stanford University School of Medicine, 1070 Arastradero Road, Suite 300, Palo Alto, CA 94304, USA; 2 Kaiser Permanente Center for Health Research, 3800 N. Interstate Ave., Portland, OR, 97227, USA

**Keywords:** Cigarette smoking, Adolescent, Menthol, African American

## Abstract

**Background:**

African Americans are disproportionately exposed to cigarette advertisements, particularly for menthol brands. Tobacco industry documents outline strategic efforts to promote menthol cigarettes to African Americans at the point of sale, and studies have observed more outdoor and retail menthol advertisements in neighborhoods with more African-American residents. Little research has been conducted to examine the effect of this target marketing on adolescents’ recognition of cigarette brand advertising and on smoking uptake. To our knowledge, this is the first study to examine racial differences in brand recognition and to assess the prospective relationship between brand recognition and smoking uptake.

**Methods:**

School-based surveys assessing tobacco use and environmental and social influences to smoke were administered to 6th through 9th graders (ages 11 to 15) in an urban and racially diverse California school district. The primary outcome for the cross-sectional analysis (n = 2,589) was brand recognition, measured by students’ identification of masked tobacco advertisements from the point of sale. The primary outcome for the longitudinal analysis (n = 1,179) was progression from never to ever smoking within 12 months.

**Results:**

At baseline, 52% of students recognized the Camel brand, 36% Marlboro, and 32% Newport. African-American students were three times more likely than others to recognize Newport (OR = 3.03, CI = 2.45, 3.74, p < 0.01) and less likely than others to recognize Marlboro (OR = 0.60, CI = 0.48, 0.73, p < 0.01). At follow-up, 17% of never smokers reported trying smoking. In this racially diverse sample, brand recognition of Camel and Marlboro did not predict smoking initiation. Regardless of race, students who recognized the Newport brand at baseline were more likely to initiate smoking at follow-up (OR = 1.49, CI = 1.04, 2.15, p < 0.05) after adjusting for shopping frequency and other risk factors.

**Conclusions:**

The study findings illustrate that African-American youth are better able to recognize Newport cigarette advertisements, even after adjustment for exposure to smoking by parents and peers. In addition, recognition of Newport cigarette advertising predicted smoking initiation, regardless of race. This longitudinal study contributes to a growing body of evidence that supports a ban on menthol flavored cigarettes in the US as well as stronger regulation of tobacco advertising at the point of sale.

## Background

African Americans are disproportionately exposed to cigarette advertisements [1], especially for cigarettes of the menthol variety [2]. Tobacco industry documents outline marketing strategies that promote menthol cigarettes to African Americans in the retail environment [3-7], and there are more outdoor and retail menthol advertisements in neighborhoods with more African-American residents [8-11]. A recent study examining advertising for menthol and non-menthol cigarettes near California high schools demonstrated that for every 10 percentage point increase in proportion of African-American students, the proportion of menthol advertising increased by 5.9 percentage points [12].

Several studies have demonstrated that youth are repeatedly exposed to cigarette marketing in the retail environment [13,14]. Youth report seeing cigarette marketing in stores [15] and are exposed to 325 brand impressions per week [16]. Stores where youth frequently shop display more cigarette advertisements than other stores within the same community [14] and corroborating evidence suggests that frequent exposure to such advertising is linked to smoking uptake [17-20].

Cigarette brand recognition has been examined as an indicator of youth exposure to tobacco marketing [21-26], but few studies specifically compare racial groups, nor recognition of menthol with non-menthol brands. One cross-sectional study of California eighth graders reported that Asian-American and European-American students were more likely to recognize advertisements for Marlboro and Virginia Slims than African-American or Latino students [25]. In two longitudinal studies, adolescents’ recognition of cigarette brands predicted smoking initiation among youth, however, neither brand- nor menthol-specific findings were reported [22,24].

Given evidence about African American and youth exposure to tobacco marketing, the present study seeks to better understand the relationship between race, brand recognition, and smoking uptake. This paper has two goals: 1) to examine racial differences in brand-specific recognition and 2) to assess the prospective relationship between brand-specific recognition and smoking uptake.

## Methods

The Survey of Teen Opinions about Retail Environments (STORE) was a longitudinal, school-based study that assessed tobacco use and environmental and social influences to smoke. Methods for this study are similar to those described in previous research in Tracy, California [14,16]. The current study was conducted in a more urban community, Vallejo, California (population 116,760), located 30 miles northeast of San Francisco. Although household median income is similar to the statewide value ($61,481 in Vallejo vs. $60,883 in the state), Vallejo is one of the nation’s most racially balanced communities, with a substantially higher proportion of African-American residents than in California as a whole (23.7% vs 6.7%) [27].

### Samples

Paper-and-pencil surveys were administered to 6-8th graders in the Vallejo school district’s four middle schools in the spring of 2006. Two follow-up surveys were conducted 12 months and 24 months after initial baseline for 7-9th graders and 8-10th graders, respectively. All students were eligible to participate in the first two years, therefore the 2007 survey functioned as both a follow-up for the 2006 participants and a baseline survey for new participants. Only students who had completed at least one baseline survey (either in 2006 or 2007) were eligible to participate in the 2008 follow-up and no new participants were recruited.

Surveys were conducted in courses that were required by all students, but these courses varied by school. Active parental consent and student assent were obtained using a protocol approved by Stanford University’s Administrative Panel on Human Subjects. In the first year, students were offered a $2 incentive to return a parental consent form, regardless of positive or negative permission. Of the 3,482 eligible students in the district, 48% of students returned a consent form. Although the incentive was increased to $5 in the second year, the return rate was similar (50%).

This paper reports analyses for both a cross-sectional sample and a longitudinal cohort (retention rate: 68%). The cross-sectional analysis (n = 2,589) combines all participants with valid baseline data for either 2006 (n = 1,647) or 2007 (n = 942). The longitudinal analysis includes participants who were never smokers at their respective baseline and reported valid data for smoking status one year later. Only 12-month follow-up data (2007) were used for those students who participated in all three surveys. Of the 2,589 students who completed a baseline survey, 1,905 reported having never tried smoking, and 1,179 of those students reported their smoking behavior at follow-up. Of the 726 never smokers that were lost to 12-month follow-up, 39% moved out of the district, 56% did not return a parental consent form for the follow-up survey, and 5% were absent on the survey day. This includes 141 students who had a 24-month follow-up but did not have a 12-month follow-up. Attrition analyses used chi-square tests for categorical variables and independent sample t-tests for numeric variables to compare all baseline covariates for the longitudinal sample (n = 1,179) and the never smokers who were lost to follow up (n = 726).

### Measures

Tobacco retailers in the study community were identified by state licensing records, and trained coders completed observations of cigarette advertising in a census of stores (n = 102, response rate = 94%) in 2006. Following procedures described in our previous studies [14,28], advertisements were counted and categorized by flavor (menthol, non-menthol, or both), by brand (Camel, Marlboro, Newport, or other), and by location (exterior, interior). For each store, we computed menthol share of voice, defined as the proportion of all cigarette advertisements in a store that featured any menthol brands.

The primary outcome for the cross-sectional analysis was brand recognition. To develop a measure of brand recognition, digital photographs of retail tobacco advertisements from the study community were altered. All brand names were removed but slogans and pack imagery were retained. Students saw the three cigarette advertisements in a fixed order: Newport, Camel, then Marlboro. These brands were selected because of their popularity among youth [29]. The advertisements showed the menthol varieties of Newport and Camel and the non-menthol variety of Marlboro. To assess advertising awareness students were first asked if they had ever seen the advertisement. They were then asked to write the name of the brand as free text. Recognition was assessed by coding these responses and misspellings were credited if they contained at least one correct syllable or approximated the phonetic pronunciation.

The primary outcome for the longitudinal analysis was progression from never to ever smoking. Smoking behavior was assessed at baseline and follow-up using the item “Have you ever tried smoking a cigarette, even one or two puffs?” We did not examine current smoking because the incidence of smoking (in the previous 30 days) was 5%.

Baseline characteristics that could confound the relationships between race, brand recognition, and smoking initiation have been described elsewhere [16]. In brief, students answered three questions about how many times they visited convenience, small market, and liquor stores each week, and these responses were combined as an overall shopping frequency measure (sum of visits per week for the three store types). Other risk factors for smoking were self-reported grades in school, recoded into 0.0 to 4.0 grade-point average (GPA), unsupervised days per week after school, and risk-taking propensity [30]. Social influences to smoke were assessed by asking about how many smokers live at home and how many of four best friends smoke; both were recoded into any versus none. Socio-demographic items included gender, race, ethnicity, and grade level. Race was coded to compare African-American students versus all other races and ethnicity was coded to compare Hispanic students to non-Hispanic students regardless of race. All analyses included an indicator for whether the baseline data came from 2006 or 2007.

#### Cross-sectional analysis

Three hierarchical generalized linear models (HGLMs) were estimated to examine recognition for each of three cigarette brands while accounting for clustering of students within schools. In each model the intercept randomly varied across the seven study schools and population average estimates were computed. The model adjusted for smoking status, shopping frequency, other risk factors for smoking, and socio-demographics. To facilitate interpretation, shopping frequency and risk-taking propensity were standardized, but GPA and unsupervised days after school were not. Covariates were fixed and non-robust standard errors were used because there were only seven schools in the analysis.

#### Longitudinal analysis

HGLMs were used to test whether brand recognition at baseline predicted smoking initiation at follow-up. Three models were estimated to examine recognition separately for each brand. Each model was adjusted for all covariates included in the cross-sectional analyses. Baseline smoking status was not a covariate because the longitudinal cohort was comprised of only never smokers at baseline. All HGLM analyses were performed using HLM6.0.

## Results

### Store observations

Tobacco retailers contained an average of 3.6 exterior ads (SD = 5.1) and 22.2 interior ads (SD = 25.9) for cigarettes. On average, the proportion of cigarette ads that featured a menthol brand (menthol share of voice) was 36.9% (SD = 18.3), which was higher than the average for the state (25.7% (SD = 26.1) [12]. The proportion of stores with any exterior advertisements for the three brands examined in the school-based surveys was 5.9% for Camel menthol, 38.2% for Marlboro non-menthol, and 33.3% for Newport. The proportion of stores with any interior ad for these brands was 22.5% for Camel menthol, 93.1% for Marlboro non-menthol, and 55.9% for Newport.

### Cross-sectional sample

Table 1 describes the cross-sectional sample and longitudinal cohort. The racial/ethnic distribution of the sample reflects the student population in the school district at the time of data collection [31]. At baseline, the prevalence of ever smoking for the entire sample was 26%, which was higher than prevalence estimates for California 7th graders in statewide school-based surveys [27]. Among African-American students, the prevalence of ever smoking was 28% and among other students it was 25%, which was not significantly different (p = 0.08). On average, students reported visiting stores almost four times per week (SD = 4.0) and African-American students reported more frequent visits compared to non-African-Americans (p < 0.01). Smoking at home also differed by race and ethnicity. The prevalence of at least one smoker at home was higher among African-American students than among others (46.8% vs. 40.6%, p < 0.01); the prevalence of home smoking was lower among Hispanic students than among non-Hispanics (37.5% vs 45.2%, p < 0.01).

**Table 1:** Sample characteristics of cross-sectional sample at baseline and never smokers at follow-up, Vallejo, CA

**Characteristics at baseline**	**Cross-sectional analysis (n = 2,589)**		**Longitudinal analysis (n = 1,179)**	
	**Variable**	**Sample**	**Variable**	**Sample**
	**n**	**% or M (SD)**	**n**	**% or M (SD)**
**Socio-demographics**				
Gender (Male)	2568	44.7%	1170	40.5%
Race	2589		1179	
African American		31.4%		31.8%
Asian/Pacific Islander		30.5%		37.7%
White		18.8%		23.1%
Other		19.3%		7.5%
Ethnicity (Hispanic)	2589	33.8%	1179	36.6%
Grade level	2578		1177	
6		18.5%		19.3%
7		36.7%		38.3%
8		31.5%		27.4%
9		13.4%		15.0%
Baseline survey year	2589		1179	
2006		63.7%		58.1%
2007		36.3%		41.9%
**Retail tobacco marketing exposure**				
Shopping frequency (visits per week)	2584	3.96 (4.03)	1177	3.27 (3.64)
Brand recognition				
Camel (menthol)	2559	52.2%	1166	48.6%
Marlboro	2559	36.1%	1168	34.4%
Newport (menthol)	2572	32.0%	1174	25.9%
**Other risk factors for smoking**				
Grade-point average	2529	2.52 (1.11)	1153	2.73 (1.05)
Unsupervised days after school	2573	2.47 (2.04)	1177	2.29 (2.05)
Risk-taking propensity	2577	2.41 (1.04)	1174	2.18 (0.92)
At least 1 smoker at home	2571	42.7%	1176	37.7%
At least 1 friend smokes	2577	34.4%	1178	25.6%
Ever smoked, at least a puff	2568	25.8%	Excluded from analysis	

Note: Shopping frequency is sum of visits per week for three store types (convenience, small market, and liquor).

**Figure 1:**
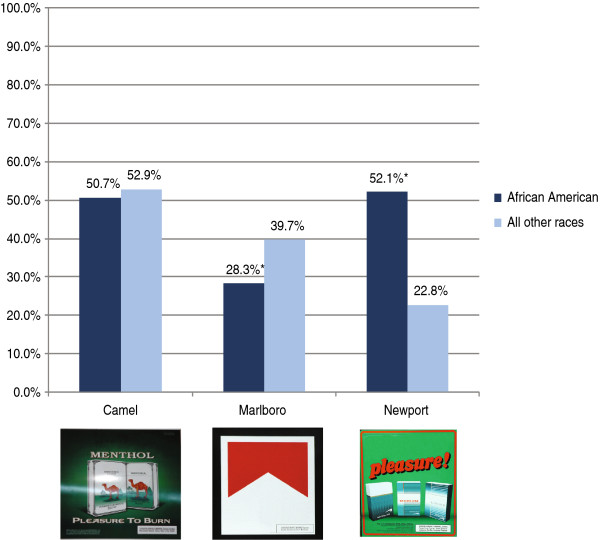
Brand recognition by African-American students versus all other races. *chi-square test p < 0.01. Note: Values are observed.

At baseline, the proportion of students who reported having seen the ads was 81% for Camel, 65% for Marlboro, and 66% for Newport. Fewer students could provide the brand name: 52% for Camel, 36% for Marlboro, and 32% for Newport. As shown in Figure 1, a significantly greater proportion of African Americans recognized the Newport brand than other students. Conversely, a significantly smaller proportion of African American students recognized Marlboro than other students. After adjusting for shopping frequency, other risk factors for smoking, and socio-demographics, the association of race and brand recognition persisted. The odds of recognizing the Newport brand was three times higher for African-American students than other students (OR = 3.03, 95% CI = 2.45, 3.74, p < 0.01) while African-American students were significantly less likely than others to recognize the Marlboro brand (OR = 0.60, 95% CI = 0.48, 0.73, p < 0.01). There were no significant racial differences in recognition of the Camel brand. Hispanic students were less likely than others to recognize Newport (OR = 0.73, 95% CI = 0.58, 0.90, p < 0.01). Other significant predictors of recognition for all three brands were living with a smoker and risk-taking propensity. In addition, older students and those who had ever smoked were more likely to recognize Newport and Camel (data not shown).

### Longitudinal cohort

Never smokers who were lost to follow-up were more likely to be boys (50.3% vs 40.5%; p < 0.01), Hispanic (36.6% vs 26.9%; p < 0.01), younger (grade level 7.2 vs 7.4; p < 0.01), and to report lower grades (GPA 2.5 vs 2.7; p < 0.01) and more store visits (3.9 vs 3.3; p < 0.01). African American youth were not more likely than other races to be lost to follow-up. No differences were observed for the other covariates, including household smoking, peer smoking, unsupervised days after school, or risk-taking propensity. In addition, never smokers who were lost to follow-up did not differ from the analysis sample on brand recognition measures (Camel p = 0.40, Marlboro p = 0.22, Newport p = 0.20).

The incidence of smoking initiation at follow-up was 17% and a greater proportion of African-American students initiated smoking than other students (22% vs 15%; p < 0.01). In an unadjusted HGLM, recognition of Newport predicted smoking initiation (p < 0.01) but neither Camel nor Marlboro recognition had a significant relationship with smoking initiation (p = 0.10 and p = 0.18, respectively; data not shown).

Table 2 presents the odds ratios and confidence intervals from three HGLMs predicting smoking initiation. Each model has a brand-specific predictor for recognition and is adjusted for all variables listed in the table. The odds of smoking initiation increased by 49% for students who recognized the Newport brand at baseline. Brand recognition for Camel or Marlboro did not predict smoking initiation at follow-up after adjusting for shopping frequency and other risk factors. Other significant risk factors for smoking initiation were race, ethnicity, risk-taking propensity, and exposure to household and peer smoking. Being African American was a significant risk factor for smoking initiation in two of the adjusted models; however, Newport brand recognition explained some of the variance attributed to that racial difference. Tests of an interaction examined whether the impact of brand recognition on smoking initiation was greater for African-American students but it was not statistically significant. In all three adjusted models, being Hispanic was also a risk factor for smoking initiation, but tests of an interaction for ethnicity with brand recognition were not statistically significant.

**Table 2:** Predictors of smoking initiation at 12-month follow-up, Vallejo, CA

	**Brand-specific predictor**					
	**Model 1**		**Model 2**		**Model 3**	
	**Camel (menthol) (n = 1,123)**		**Marlboro (nonmenthol) (n = 1,125)**		**Newport (menthol) (n = 1,131)**	
	**OR**	**95% CI**	**OR**	**95% CI**	**OR**	**95% CI**
**Retail tobacco marketing exposure**						
Recognition of brand in column	0.94	(0.68, 1.31)	1.19	(0.84, 1.66)	1.49*	(1.04, 2.15)
Shopping frequency	1.16	(0.98, 1.39)	1.17	(0.98, 1.39)	1.12	(0.94, 1.34)
**Socio-demographics**						
Male	1.10	(0.79, 1.54)	1.11	(0.80, 1.54)	1.11	(0.80, 1.55)
Race (African American)	1.59*	(1.10, 2.31)	1.62*	(1.11, 2.35)	1.46	(0.99, 2.15)
Ethnicity (Hispanic)	1.61**	(1.14, 2.27)	1.58**	(1.12, 2.24)	1.61**	(1.14, 2.28)
Grade level	0.91	(0.74, 1.12)	0.91	(0.74, 1.12)	0.92	(0.74, 1.13)
Baseline year	1.07	(0.73, 1.56)	1.07	(0.73, 1.57)	1.09	(0.75, 1.60)
**Other risk factors for smoking**						
Grade-point average	0.92	(0.78, 1.07)	0.91	(0.78, 1.06)	0.91	(0.78, 1.07)
Unsupervised days after school	1.01	(0.93, 1.09)	1.01	(0.93, 1.09)	1.01	(0.93, 1.09)
Risk-taking propensity	1.35**	(1.13, 1.62)	1.33**	(1.11, 1.60)	1.35**	(1.12, 1.62)
At least 1 smoker at home	1.60**	(1.12, 2.22)	1.58**	(1.14, 2.20)	1.51*	(1.08, 2.11)
At least 1 friend smokes	1.46*	(1.03, 2.08)	1.44*	(1.01, 2.05)	1.36	(0.95, 1.94)
Intercept	0.26	(0.04, 1.51)	0.23	(0.04, 1.42)	0.21	(0.03, 1.28)

*p < 0.05, **p < 0.01.

An ancillary analysis imputed missing data for the subset of baseline never smokers (n = 141) who had follow-up data at 24 months but not at 12 months. When self-reported smoking at 24-month follow-up was substituted for missing data at 12-month follow-up, the association of Newport brand recognition with smoking initiation persisted (data not shown).

## Discussion

The current study is the first we are aware of to examine relationships between race, cigarette brand recognition, and smoking initiation. The findings document a racial difference in adolescents’ cigarette brand recognition but observed no racial differences in the impact of brand recognition on smoking initiation. Compared to other students, African Americans were less likely to recognize Marlboro and more likely to recognize Newport, which is consistent with evidence that African-American youth are disproportionately exposed to advertising for menthol cigarettes. Regardless of race, recognition of Newport predicted smoking initiation, which is consistent with other suggestions that menthol advertising encourages youth smoking [2,5,6,32].

Overall, Camel was more recognizable than Marlboro and Newport which is consistent with previous research [25]. Although Newport is the leading menthol brand and its market share is nearly eight times greater than Camel’s menthol product (9.8% vs 1.3%) [2], any Camel ad is likely more recognizable than other brands because its icon is a literal representation of the brand name. The current study documents a racial difference in cigarette brand recognition that may help to explain the popularity of Newport among teen smokers in the US, particularly among African Americans. Among US smokers in middle school and high school (ages 12–17), Newport is the second most popular brand overall and more popular among African Americans than any other racial/ethnic group [29].

Our findings expand on a previous study by Unger and colleagues [25] which reported that race and smoking status were significant correlates of brand recognition for cigarette advertisements in magazines. However, that cross-sectional study could not clarify whether brand recognition precedes smoking initiation or vice versa. In this longitudinal study, recognition of Newport predicted a higher likelihood of smoking initiation, adjusting for other risk factors, such as the presence of a smoker at home and exposure to peers who smoke.

Newport was the only brand for which recognition was predictive of smoking initiation at follow-up, which may be attributable to the youthful appeal of its advertising and its prevalence in African-American neighbourhoods [2]. Our previous study documented a greater availability of Newport price promotions near California high schools with a higher proportion of African-American students and in school neighborhoods with a higher proportion of residents ages 10-17 [12]. In the current study, Vallejo stores contained a greater proportion of cigarette advertising for menthol brands than the average proportion for stores in California. This may explain in part why the prevalence of ever smoking at baseline was higher for all racial/ethnic groups, and the incidence of smoking initiation was higher for African-American and Hispanic students.

Initial low response rates (48% in year 1, 50% in year 2) and subsequent loss to follow-up are limitations of this study but this bias is conservative and reduces the power to detect associations. Because students lost to follow-up reported significantly more store visits at baseline, it is possible that this study underestimated brand recognition among youth as well as its influence on smoking initiation.

Another limitation of this study is that students responded to just one visual representation of each brand. Future research should assess recognition using multiple examples of the menthol and non-menthol varieties from several brand families in order to determine if exposure to advertising for a specific brand or flavor is more influential. Recognition measures could be adapted to measure flavor-specific as well as brand-specific marketing exposure in order to better document the impact of tobacco product marketing among population subgroups. Linking data from the retail environment with school survey data has provided valuable insight into the relationship between point-of-sale advertising exposure and adolescent smoking [11,14,23,28]. The role of brand recognition as a mediator of this relationship would benefit from further investigation.

## Conclusions

The racially diverse sample and the longitudinal design of this study allow for new insights into the impact of targeted marketing. The significantly better recognition of Newport brand advertising among African-American youth is noteworthy. In the current study, recognizing the Newport brand was a risk factor for smoking initiation regardless of race, suggesting that stronger enforcement of the Master Settlement Agreement is needed to protect all youth from tobacco marketing at the point of sale. In addition, the study findings are consistent with a predicted benefit to adolescent health from banning menthol cigarettes and further regulating tobacco advertising at the point of sale.

## Competing interests

The authors declare that they have no competing interests.

## Authors’ contributions

AD participated in the design of the study and its coordination, and drafted the manuscript. JD drafted the manuscript. NS participated in the design of the study and performed the statistical analysis. SF participated in the design of the study. LH conceived of the study, participated in its design, and drafted the manuscript. All authors read and approved the final manuscript.

## Implications and contributions

African Americans are disproportionately exposed to cigarette advertisements, particularly for menthol brands. Findings from this longitudinal study suggest the detrimental effect of exposure to marketing for Newport cigarettes on smoking uptake by youth. The results are consistent with a predicted health benefit of banning menthol cigarettes in the US.

## Pre-publication history

The pre-publication history for this paper can be accessed here:

http://www.biomedcentral.com/1471-2458/13/170/prepub
